# Supplementary material for: West Nile Virus Meningoencephalitis—A Consideration for Earlier Investigation
Source: Reports (MDPI). 2024 Mar 27;7(2):23. doi: 10.3390/reports7020023 (PMC12225240; doi:10.3390/reports7020023)
Supplement: Supplementary file 1 [file reports-07-00023-s001.zip › reports-2909313-supplementary.pdf]

Supplementary Materials

Case Report

West Nile Virus Meningoencephalitis – A Consideration for Earlier Investigation

David Burns [1,*], Zachary Vinton [1], Min Kyung Chung [2], and Johnny Cheng [1]

[1] Rocky Vista University College of Osteopathic Medicine, Englewood, CO, United States
[2] Rocky Vista University College of Osteopathic Medicine, Ivins, UT, United States
* Correspondence: David Burns, david.burns@rvu.edu

Methods

In this report, we follow a case of an elderly female with neuroinvasive WNV in order to answer the question: does this case of neuroinvasive WNV show unique features which could inform future clinical decision making? Data collection was completed after the patient was discharged through a review of their electronic medical record for events which occurred while they were admitted to the hospital. A literature search was performed to enhance our understanding of neuroinvasive WNV as well as the various features of our patient's disease that were considered typical or atypical. To accomplish this, we performed a literature search on Pubmed with the following keywords or terms: "West Nile Virus" and "Neuroinvasive" to find published case reports and literature reviews, where the full text was available online. The database yielded a total of about 9,948 results. We subsequently narrowed our search using the terms "ocular", "neurologic", "cardiac", and "movement" to further compare our patient's presenting features with others in the literature. This yielded a total of 40 results. Following the literature search, we also reviewed the most current guidelines and statistics from the Center for Disease Control for general information on the disease.

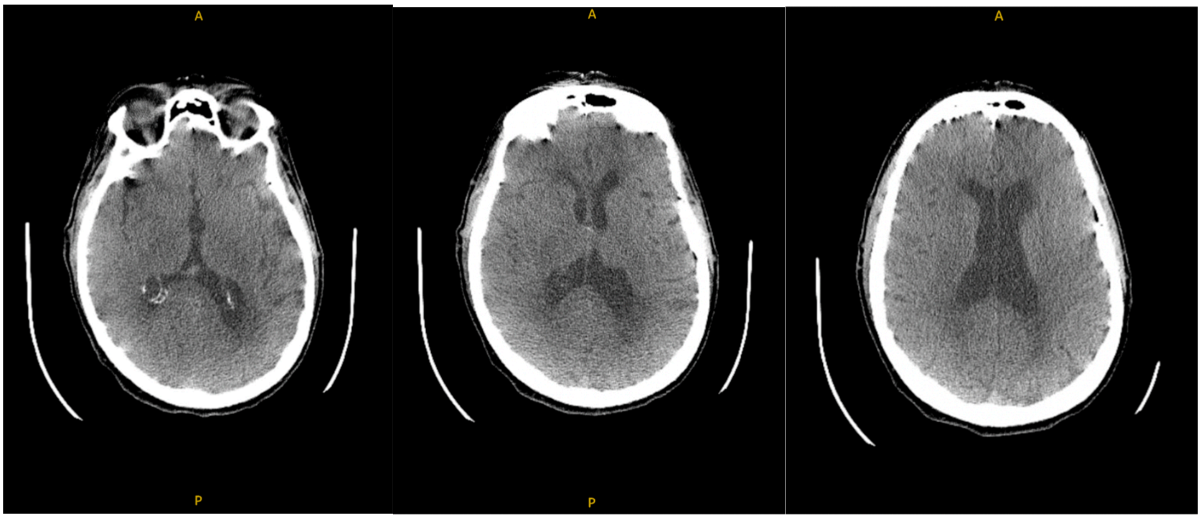

Figure S1. Computed Tomography (CT) Head Without Contrast. No acute intracranial abnormality with nonspecific white matter changes, most consistent with chronic microangiopathic change.

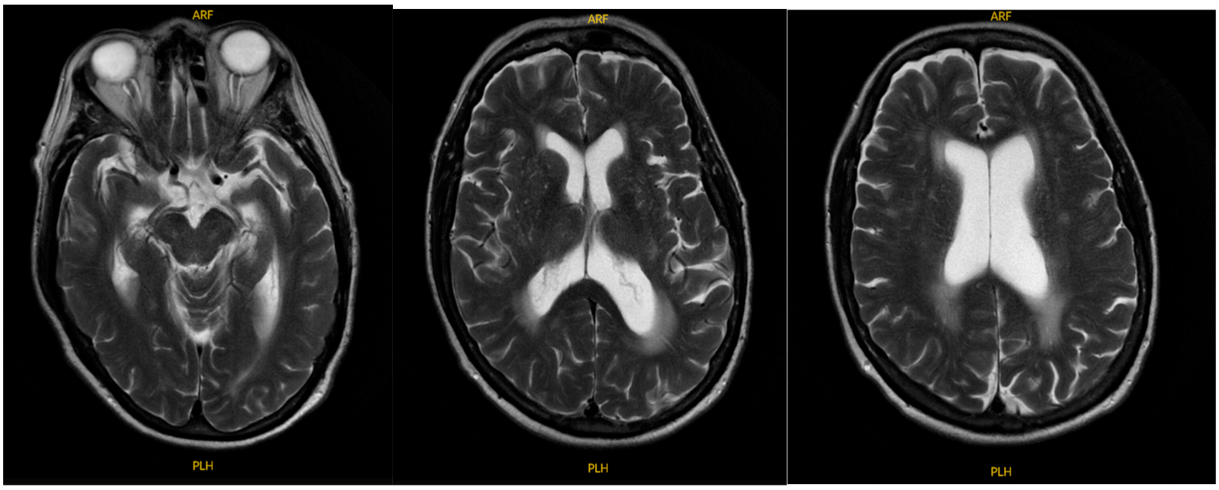

Figure S2. Magnetic Resonance Imaging (MRI) Head Without Contrast. No acute infarct, hemorrhage, or mass. Roughly age commensurate parenchymal volume loss. Mild to moderate chronic microvascular changes.
